# Ageing and Mental Health in Canada: Perspectives from Law, Policy, and Longitudinal Research

**DOI:** 10.1007/s12062-022-09389-z

**Published:** 2022-08-19

**Authors:** T. D. Cosco, C. Randa, S. Hopper, K. R. Wagner, J. Pickering, J. R. Best

**Affiliations:** 1 Gerontology Research Centre, Simon Fraser University, Vancouver, BC Canada; 2 Department of Gerontology, Simon Fraser University, Vancouver, BC Canada; 3 Oxford Institute of Population Ageing, University of Oxford, Oxford, UK; 4 Department of Government and International Relations, University of Sydney, Sydney, NSW Australia; 5 Department of Psychiatry, University of British Columbia, Vancouver, BC Canada

**Keywords:** Mental health, Ageing, Mental health law, Canadian policy, Longitudinal research

## Abstract

Canada is a relatively young, geographically-diverse country, with a larger proportion of the population aged over 65 than under 15. Increasing alongside the number of ageing Canadians is the number of older adults that live with mental health challenges. Across the life course, one in five Canadians will experience a mental health disorder with many more living with subclinical symptoms. For these individuals, their lived experience may be directly impacted by the contemporary laws and policies governing mental illness. Examining and reviewing the historical context of mental health and older adults, we provide insights into the evolving landscape of Canadian mental health law and policy, paternalistic roots in the infancy of the country, into modern foci on equity and diversity. Progressing in parallel to changes in mental health policy has been the advancement of mental health research, particularly through longitudinal studies of ageing. Although acting through different mechanisms, the evolution of Canadian mental health law, policy, and research has had, and continues to have, considerable impacts on the substantial proportion of Canadians living with mental health challenges.

## Introduction

In 2016, the Canadian census results revealed that there were more older adults than children, for the first time in census history. Reporting a record 5.9 million Canadians aged 65 and older, Statistics Canada noted that this figure exceeded the 5.8 million Canadians aged 14 and under (Statistics Canada, 2017). Driving the Canadian demographic shift, as has been observed elsewhere in the Western world, have been the post-World War II baby boom and bust, which lasted from 1946 to the mid-1960s and the mid-1960s to the early 1970s. Another contributing force to the increase in the proportion of older adults has been the combination of a decrease in fertility, along with an increase in life-expectancy for both males and females (Statistics Canada, 2021). The number of persons aged 65 and over increased 14.1% between 2006 and 2011 to nearly 5 million. This rate of growth was higher than that of children aged 14 and under (0.5%) and people aged 15 to 64 (5.7%). As more of the baby boom generation reaches the 65-year threshold, they have greatly outpaced the numbers of children being born; this has resulted in older persons representing 18.5% of the population and children just 15.7% (Statistics Canada, 2021).

Considering Canada’s vast geographic diversity and nearly 10 million square kilometres of land, it is not surprising that the distribution of where ageing Canadians live is not uniformly distributed. The Eastern provinces, notably the Maritimes (i.e., Newfoundland and Labrador, Prince Edward Island, Nova Scotia, and New Brunswick), have much older populations than their western (i.e., British Columbia and Alberta), and northern (i.e., Yukon, Northwest Territories, and Nunavut), counterparts (Statistics Canada, 2021). The northern Canadian territories experience both lower life expectancy and higher rates of fertility, resulting in the youngest populations observed in Canada. Of note, Canada’s youngest territory, which was first recognized in 1999, Nunavut, also boasts the youngest population, with just 4.1% of inhabitants aged 65 years and older (Statistics Canada, 2021).

## Mental Health Disorders in Canada

Mental health disorders affect up to one in five Canadians every year (Smetanin et al., 2011). Among older adults specifically, this number decreases to approximately 17%. In survey data collected from November 2021 to February 2022, 29.5% of older Canadians reported that their mental health was either somewhat or much worse than pre-pandemic (Statistics Canada, 2022a). Though these rates are still lower than younger Canadians, older adults are a unique case. First, older adults are prone to underreporting mental health disorders (Lyness et al., 1995). Second, older adults are less likely to be referred to psychological therapy (Pettit et al., 2017). Finally, older adults are less likely to seek mental health services (Byers et al., 2012). An underserved population throughout psychiatric history, older adults living with mental health issues also face ageism within the healthcare system from clinicians and internalised stereotypes and biases toward mental illness and treatment strategies, and negative attitudes about ageing (Bodner et al., 2018).

## Mental Health Laws and Policies in Canada

Contemporary laws and policies concerning mental health may have considerable impacts in the lived experience of mental ill-health. In Canada, approaches to mental health law and policy have evolved rapidly over the last century, shaping both better or worse the ways in which individuals living with mental disorders are viewed and treated. This could range from how individuals living with mental illness are given legal sentences to how the public perceives mental illness. Laws implement justice for the people, whereas policies are enacted by governments with the aim of achieving a goal reflected by the values of society at a distinct time (Lowi, 2003). From paternalistic asylums sequestering those living with mental illness away from families and communities to the deinstitutionalization of long-term mental health facilities in the 1960s, Canada is working toward a more holistic approach to mental health service delivery, laws, and policies including the voices of individuals living with mental illness and their families and other stakeholders. Today, mental health service providers in Canada seek to honour culture, individual preference, and reconcile the stigma and mistrust of institutional care in the past with the goal of an equitable and culturally appropriate present and future.

Canada, in the tradition of other Commonwealth countries, has developed mental health laws out of legislative reform in contrast to America’s history of constitutional challenges from mental health advocates which have sought to tilt the scales of power imbalance between patients, mental health professionals, and governments (Gordon & Verdun-Jones, 1986). Advocacy and exercising agency for older adult mental health services and legal protection is a relatively new concept, with policies and practices under the jurisdiction of provincial governments under the same healthcare umbrella including mental health (British Columbia Ministry of Health, 2000; Elderly Mental Health Care Working Group, 2002; Kaiser, 2009).

### An Overview of Mental Health Law and Policy in Canada

Mental health laws and services are provincially regulated through 13 different mental health acts across the 10 Canadian provinces and 3 territories (O'Reilly & Gray, 2014). Legislation at the provincial level can create significant variance between mental health laws and strategies; however, all Canadian laws must uphold the Canadian Charter of Rights and Freedoms (O'Reilly & Gray, 2014). If believed to violate charter rights, laws can be challenged in the Supreme Court of Canada with landmark cases often ushering in new or amended laws and policy reform. Bodner et al. (2018) draw attention to the gap in literature exploring the relationship between ageism and barriers to older adults accessing mental health services. The following section aims to provide an overview of the history and evolution of major movements in Canadian mental health law and the resulting implications for older adults.

#### Paternalism: 1800s–1950s

Historically, Canada’s treatment of people living with mental illness is not dissimilar to the inhumane conditions in asylums across North America and Europe in the 19^th^ and into the twentieth century (Reaume, 1997, 2002). This shameful legacy of paternalism, isolation, abuse, overcrowding, and underfeeding still negatively influences the inclusion and social justice of Canadians living with mental illness as stigma persists (Ontario Human Rights Commission, 2015). Paternalistic mental health care approaches emphasise the importance of protecting society from those with mental illness and treatment through isolation to reduce harm to self and others, especially through medical care (Tyhurst et al., 1963). Consequently, individuals living with mental illness during this era would have experienced considerable abuses at the hands of the legal system alongside broader stigmatisation.

Laws protecting the rights and freedoms of those living with mental illness were not a priority until the mid-1900s, ‘limited legalism’ described the hands-off approach to the access and regulation of services for the mentally ill (Gordon, 1988). Although there have been glimpses into experiences and perceptions of older adult mental health through literature and historical artefacts, geriatric psychiatry was not established as a field of study until the 1900s (Le Clair & Sadavoy, 1998), entering a new era, based on an organic understanding of the brain founded in biology and neuropathology, further legitimising the field to align with mainstream medicine (Sussman, 2018). The World Wars brought about a boom in social services, specifically psychiatric care in general hospitals and community settings, as attitudes changed regarding who could experience mental health issues considering the trauma of war (Sussman, 2018). During this period of 'medicalization', governments began to supervise medical practitioners more judiciously and increased funding for medical research while beginning to form a strategy for more unified social health policies (Gordon, 1988). With scientific advances and evolving societal perceptions, a movement began for voluntary commitment as well as volunteerism to bridge the harsh divide between the community and mental health care facilities and treatment hospitals (Sussman, 2018). The Canadian National Committee for Mental Hygiene, later to be renamed the Canadian Mental Health Association (CMHA), was founded in 1918 and began commissioning provincial reports evaluating conditions within mental hospitals (Reaume, 2002; Sussman, 2018). With advances in medical and scientific knowledge of mental illness and widespread societal changes, Canadian mental health care service delivery was on the precipice of a revolution at the dawn of the 1960s.

#### Deinstitutionalization: 1960s–1980s

Displays of human agency through activism in the 1950s brought about another period of law reform in Canada in the 1960s characterised by 'enhanced medicalisation' where psychiatry further harmonised with mainstream medicine as a biologically based treatment where both patients and practitioners have rights (Gordon, 1988). Adopting a policy of deinstitutionalization, Canada began a rapid closure of long-term psychiatric facilities in favour of community-based supports and services for greater autonomy and social connection (Reaume, 2002). This period of deinstitutionalization also influenced older adult care policies, with a return to more community-based care with the Ageing-in-Place movement (Wiles et al., 2012). The goal was to create a system where patients could be admitted to hospital for short-term treatment when unwell, then return to the community for ongoing support while fostering independence (Wasylenki, 2001). Not entirely unsuccessful, this movement created a divide in quality of life between those with less severe mental health struggles being more likely to utilise psychiatric services in general hospitals, but those with serious and persistent mental illness still needed to live in provincial psychiatric facilities with resources and a resurgence of stigma (Reaume, 2002; Sussman, 2018; Wasylenki, 2001). Although well-intentioned, the transition from institutional to community care was not systematically supported by the government for long-term success, marked by a lack of consideration for affordable and safe housing, access to reliable care within the community after discharge, and accountability of conditions for those with serious and persistent mental illness still living in long-term psychiatric care facilities (Ontario Human Rights Commission, 2015).

In 1961, Canada reached a turning point for the organisation and supervision of mental health services through the appointment of the Royal Commission on Health Services (Ford, 1964). In the first report of its kind, Ford summarised the Commission’s recommendations for the betterment of healthcare services for all Canadians through a national health policy and program for health services, advancement of healthcare staff training and research, as well as sustainable government financing. In the 1970s, the Canadian Psychiatric Association formed the section of Geriatric Psychiatry, with training in medical schools monitored and mandated by the Royal College of Physicians and Surgeons of Canada (RCPSC) to meet national training standards and solidify geriatrics as a sub-specialization of psychiatric medicine (Le Clair & Sadavoy, 1998). The Canadian Academy of Geriatric Psychiatry (CAGP) was formed in 1993 and provided a national platform for research, education, and advocacy for mental health in older adults (Andrew & Shea, 2010). Following years of building consensus among psychiatrists as to the key features of geriatric psychiatry subspecialty training, the RCPSC approved the CAGP application by the CAGP for subspecialty recognition in 2009. Additionally, the CAGP has been instrumental in creating educational initiatives–including the creation of the first set of evidence-based national guidelines for older adult mental health care and creation of educational programs–focused on primary psychiatric care for older adults (Andrew & Shea, 2010). The 1970s and 1980s were characterised by ‘new legalism’ in Canada, shifting economic policies influenced the delivery of mental health services not only in institutional and government-run settings such as hospitals, but also brought to the forefront the importance of regulation for community support services after admission (Gordon, 1988). By 1982, few civil mental health cases had been brought forth to enact change in Canadian mental health law; however, the Constitution Act within the Charter of Rights and Freedoms opened opportunities to challenge mental health law in the judicial tradition of the American Supreme Court (Gordon & Verdun-Jones, 1986). With the guidance of the Charter, provincial mental health acts were introduced. Notably, in 1985, the Saskatchewan Mental Health Services Act expanded upon the Charter to include legislation designed to protect mental health patients from involuntary commitment unless at risk for harm to self or others, the right to refuse treatment, and that consent must be given for diagnostic or treatment services unless in an emergency (Government of Saskatchewan, 1985). A major criticism of the Charter in this iteration was a lack of government accountability to provide resources ensuring Canadians in any province could access community-based treatment as a human right by judicial decree (Gordon & Verdun-Jones, 1986). In the years following the enactment of the Charter, a burgeoning field of mental health law emerged with close ties to the criminal justice system. As the law was analysed and reviewed, recommendations were heeded in 1987 with advocacy from the CMHA’s support for Bill 190, an amendment to the Ontario Mental Health Act which granted patients the right to choose treatment alternatives (Canadian Mental Health Association, n.d.; Kaiser, 2009). This collaboration between advocates, patients, and government marked the beginning of a new era in Canadian mental health law where the voices and experiences of a variety of stakeholders were beginning to be taken into consideration for laws and policies.

#### Introduction of Stakeholders: 1990s–2010s

Reflecting on the history of Canadian mental health law and policy summarised previously, the inclusion of clients, their families, and allies outside the medical and psychiatric profession is a relatively recent concept (Davis, 2006; McGrath & Tempier, 2003). In 1997, Health Canada introduced the inclusion of stakeholders in a report on best practices in mental health reform. Stakeholders are individuals or groups to be consulted and included in the development of public mental health policy and consist of three groups: practitioners, family members, and clients (Davis, 2006). The digital revolution offered instantaneous access to information and resources regarding mental health services, as pioneered by the CMHA’s website launch in 1999 (CMHA, n.d.). The Supreme Court of Canada aims to protect human dignity through laws set to preserve self-respect and self-worth as set out in the Charter of Rights and Freedoms (Kaiser, 2009). However, with the variance in provincial legislation and service availability and quality, there was a growing demand for a national research agenda including the recommendations of individuals with lived experience of mental illness and their care partners with support from advocacy groups and organisations to enrich recommendations for a unified approach to mental health care in Canada. An estimated 80% to 90% of older adults living in long-term care have some form of mental disorder, with approximately 50% living with a diagnosis of depression: when depression is preventable and treatable, it is imperative to not write off older adult mental illness as a normal part of the ageing process (Canadian Coalition for Senior’s Mental Health, 2009). In 2002, British Columbia’s Elderly Mental Health Care Working Group (2002) published a collection of guidelines for best practices for health authorities across the province to improve planning, design, evaluation, and delivery of mental health services specifically for older adults. These guidelines served as a benchmark for the implementation of older adults’ mental health services across the country.

#### A Equity and Diversity: 2010s–Present

Mental health legislation in Canada today seeks to harmonise individual rights and freedoms with diverse identities and cultures of the Canadian population. The Mental Health Commission of Canada’s mandate was to create the first national mental health strategy which was released in 2012 (Mental Health Commission of Canada, 2021). Even with a unified federal mental health strategy, there are still variations in legislation between provinces. An essential component of current mental health acts across provinces is the correct application of the committal process, committal criteria, and rights procedures to meet the distinctions between voluntary admission, compulsory in-patient treatment, and community treatment orders without violating human rights (O'Reilly & Gray, 2014). Mandatory outpatient treatment is perceived as a safeguard for those with severe mental illness living in the community to receive a comprehensive plan of treatment while lacking the insight to consent for their own safety and the safety of others (O’Reilly et al., 2010). The inclusion of stakeholders in the 1990s has further evolved to become more inclusive of older adults without advocates and fictive kin, or supportive people in someone’s life either providing or assisting with the coordination of care and instrumental activities of daily living (Jordan-Marsh & Harden, 2005). This shift acknowledges meaningful relationships beyond nuclear families.

As Canada enters the second decade of the new millennium, mitigating discrimination and barriers to accessing mental health care is at the forefront of policy and service delivery. In Canada’s cultural mosaic, protective associations have been observed in communities with higher ethno-cultural density; thereby supporting community mental health through access to culturally and linguistically appropriate healthcare reducing stigma or discrimination, although more research is necessary to understand the nuanced implications for older adults specifically (Emerson et al., 2021). Organizational policies, behaviour patterns, and practices that reinforce or create disadvantages for people living with mental illness are categorized as systemic discrimination (Ontario Human Rights Commission, 2015). In the spirit of advocacy, legislation detailing the mandate and duties of The Office of the Seniors Advocate was passed on March 14, 2013. The Seniors Advocate in British Columbia is tasked with monitoring and analysing services for older adults including mental health support, crafting recommendations for governments and service providers for systemic improvements, and collecting resources for those utilising the services (Government of British Columbia, 2013). Reporting to the Minister of Health, the Seniors Advocate releases an annual advocacy report examining health care, housing, income supports, community supports and transportation in relation to the efficiency, outcomes, and effectiveness for older adults in British Columbia with the participation of a community advisory including experiences and perspectives of older adults from different geographic locations, cultures, and ages (Government of British Columbia, 2013). As part of Canada’s committed to truth and reconciliation, the co-development of distinctions-based Indigenous health legislation aims to transform health service delivery through collaboration with Indigenous organisations in the design, provision, and improvement of services to and establish principles of respect and partnership increasing Indigenous-led health services (Indigenous Services Canada, 2022).

Older adults living with mental illness are at the intersections of multiple jeopardies: ageism, ableism, and the stigma associated with mental illness. Suicide is disproportionately high among older adults, as older adults account for 18% of all suicides (Segal et al., 2018). Serious persistent mental illness statistics are vague in the Canadian context, with little information and services specifically tailored for older adults. With one in five Canadians reported to experience mental illness at some point in their lifetime, mental illness is a reality for many older adults (Woods et al., 2008), a reality that is experienced within the context of contemporary laws and policies.

## An Overview of Longitudinal Mental Health Research in Canada

The lived experience of mental health is not only shaped by contemporary laws and policies, but also by contemporary best-evidence practices and treatments. Older adults’ mental health trajectories are impacted by myriad factors, from the resilience-fostering to the depression-inducing. In order to identify these factors and the ways in which better mental health can be fostered, we must look to robust sources of evidence, such as longitudinal studies.3. In contrast to cross-sectional research, which only captures data at one point in time, longitudinal research involves repeated data collection, generally using the same measurement techniques on groups of the same individuals over time. When compared with cross-sectional research, there are clear advantages. Cross-sectional research is beset by cohort effects where environmental and social factors may be conflated with personal or intrinsic ones, making the identification of directional causality treacherous (Baltes, 1968). Comparatively, longitudinal data allow for the ordering of events in time. They can also provide much richer detail, allowing for the adjusting of confounding effects and unobserved heterogeneity. Longitudinal research initially became popular to understand the effects of numerous variables on a child’s development (Sontag, 1971), but expanded over time to include development across the lifespan. Longitudinal studies are now the preferred method of identifying causes and effects in health sciences data. In studying the trajectories of older adults’ mental health, researchers have benefitted from the rich data resources of Canadian longitudinal studies.

The following is a brief synopsis of Canadian studies that have taken a longitudinal approach to studying the processes of ageing and mental health.

### Canadian Longitudinal Study on Aging: 2010–Present

The Canadian Longitudinal Study on Aging (CLSA) is a large national study of over 50,000 adults aged 45–85 at baseline (Raina et al., 2019). Baseline data collection started in 2010, with data collection occurring every three years for 20 years, or until participant death. The CLSA is comprised of two cohorts: a tracking (*n* = 21,241) and a comprehensive cohort (*n* = 30,097). The tracking cohort participates in telephone interviews whereas the comprehensive cohort undergoes face-to-face in-home interviews, as well as in-depth data collection at data collection sites across Canada. A broad range of data is collected including lifestyle factors, psychological health, social behaviour, medications, and physical assessments (Raina et al., 2009). Specific data on mental health is collected including mood disorders, depressive symptoms, post-traumatic stress disorder, anxiety, and psychological distress.

### Canadian Community Health Survey: 2001–Present

The Canadian Community Health Survey (CCHS) collects health data from a sample of Canadians over the age of 12, at the provincial, and intra-provincial levels (Statistics Canada, 2022b). From 2001–2005 data from 65,000 participants was collected every two years, with data collection taking place annually thereafter. The survey collects data regarding participants’ physical and mental health, chronic conditions, use of healthcare services, and health behaviours (Statistics Canada, 2022b). A measure of self-reported mental health is collected, along with self-reported stress, and a measure of depression.

### Longitudinal and International Study of Adults: 2011–Present

The Longitudinal and International Study of Adults (LISA) aims to understand changes to Canadian society over time (Statistics Canada, 2020). The LISA began in 2011, following approximately 34,000 Canadians over the age of 15, every 2 years. Data regarding jobs, education, health, and family is collected, with the objective to understand intergenerational connections and factors that impact families and individuals (Statistics Canada, 2020). A measure of self-reported mental health is collected, along with a questionnaire providing an overall measure of mental health.

### Canadian Study of Health and Aging: 1991–2001

The Canadian Study of Health and Aging (CSHA) was a three-wave longitudinal study of 10,263 adults ages 65 and older (Canadian Study of Health & Aging Working Group, 1994). Data was collected in 5-year increments with baseline data collection taking place in 1991. At baseline, 9,008 participants lived in the community, and 1,255 lived in institutions. Originally designed to study the epidemiology of dementia, the CSHA also produced data regarding healthy ageing, disability, frailty, and more (McDowell et al., 2001). Self-reported mental health was included in the general health section of all three waves, while additional questions regarding psychological well-being were included in the second wave.

### National Population Health Survey: 1994–2012

The National Population Health Survey (NPHS), organised by Statistics Canada, was a nine-wave longitudinal study, collecting data regarding the health of the Canadian population every two years (Statistics Canada, 2010). At baseline in 1994 the NPHS included 17,276 Canadians of all ages. The survey included questions regarding various aspects of health such as, nutrition, chronic conditions, physical activity, mental health, stress and more (Statistics Canada, 2010). Questions regarding mental and emotional well-being were included in all nine waves.

## Overarching Trends in Longitudinal Mental Health Research in Canada

### Mental health in historically marginalised groups

As noted previously, mental health legislation in Canada has been in an era focused on equity and diversity since the 2010s. This focus is seen not only in legislation, but also in the research that informs it. In analysing CLSA data, researchers have published extensively on marginalised sub-populations of older adults. Instead of seeing older adults as a homogeneous body, there is an acknowledgement of intersectional diversity. Stinchcombe et al. (2018), for example, examined the relationship between health inequities and sexuality. Their research revealed the key finding that female and male sexual minorities have greater odds of reporting mood disorders than do their heterosexual counterparts. In another study by Davison et al. (2020) investigating correlates of psychological distress among older adults, immigrant status is revealed to be strongly associated. Such research has direct bearing not just on clinical management but on broader policy and law as relates to the protection of these groups.

### COVID-19-Related Impacts on Mental Health

One salient trend in recent research is to examine the multi-faceted effects—especially mental health impacts—of the COVID-19 pandemic on individuals. This is eminently sensible given the dramatic changes to daily life encouraged—and in some cases mandated—in the early days of the pandemic and continuing to this day in various parts of the world to achieve physical distancing and reduce person-to-person transmission of SARS-CoV-2. Many cross-sectional studies and non-representative studies have been conducted to understand effects on mental health; however, the impact of the results from these studies is diminished given that pre-pandemic health can only be ascertained retrospectively, and selection bias limits the generalizability of findings. In contrast, the CLSA was well-suited to evaluate mental health and well-being in middle- and older-aged adults as the COVID-19 pandemic necessitated drastic changes to daily life starting in March 2020. The primary reason for this is that the CLSA cohorts were formed using population-based sampling and pre-existed the pandemic. The CLSA COVID-19 Questionnaire Study launched rapidly in April of 2020 to better understand mental health impacts, stressors, and pandemic-related daily experiences among participants in the larger CLSA study. An initial questionnaire was completed in April–May 2020, followed by monthly questionnaires and a final exit questionnaire September–December 2020. This design allows for an assessment of pre-pandemic mental health, mental health shortly after the WHO declared the COVID-19 pandemic and governments instituted initial lockdowns, and mental health following the initial waves of pandemic but prior to the wide scale availability of vaccines. Results suggested a near doubling in the odds of moderate to clinically relevant levels of depressive symptoms in comparison the pre-COVID period between 2015 and 2018 (Raina et al., 2021). Depressive symptoms appeared to exacerbate over the initial waves of the pandemic through the end of 2020 before vaccines were widely available. Various risk factors have been identified for a disproportionate negative impact on depression and well-being among Canadian adults, including lower socio-economic status, pre-pandemic multi-morbidity, and interestingly, being middle-aged (45–55 years) as compared to older aged (75 years +) (Raina et al., 2021). Other findings have demonstrated the negative association between stressors experienced during the pandemic and concurrent mental health, such as loss of income, increased caregiver burden, and family conflict (Raina et al., 2021; Wister et al., 2022a, b). Still other research has shown that Canadian adults who reported a diminished ability to engage in social and physical activities because of the pandemic show added risk for high levels of depressive and anxiety symptoms (Cosco et al., 2021).

### Environmental Influences on Mental Health

Satellite imagery along with increased computational resources has allowed researchers to generate highly detailed maps of the environments in which individuals live. Such data will enrich longitudinal studies by allowing researchers to evaluate research questions, such as: “What is the expected impact of an objective environmental exposure on changes in an outcome of interest within individuals over time?” The Canadian Urban Environmental Health Research (CANUE) provides a common platform of standardised environmental exposures that health data organisations link to ongoing studies for the benefit of the Canadian research community (https://www.canue.ca). Data include measures of air quality, green and blue spaces, weather, and socio-economic indices of the neighbourhood. Such data has enriched the CLSA by allowing researchers to explore associations between objective environmental exposures on the mental health of middle- and older-aged adults. One early study has suggested that greater urban greenness is associated with superior mental health in the first wave of the CLSA (Abraham Cottagiri et al., 2022). Future research trends in this area will likely: consider additional aspects of the environment as a contributor to mental health; evaluate whether there are longitudinal associations; and identify potential mechanisms that link the environment to mental health, such as behaviours (Klicnik et al., 2022), social connections (Gan et al., 2022), and disease (Grant et al., 2021).

### Modelling of Change and Dynamic Connections

Longitudinal studies in Canada—especially the CLSA—are well-suited to utilise and evaluate sophisticated models of change in mental health. By the end of the CLSA, participants will be followed for at least 20 years, or until death, with data collection occurring every three years (as of July 2022, data from the baseline and first two follow-up assessments are available for analysis). With a reasonable number of assessments of mental health (e.g., depressive symptoms) per participant (i.e., 6–7), analysts will be able to use these data to address important questions: what trajectories do mental health and well-being follow within people? Do the trajectories differ by age, gender, or any other characteristics? How much do mental health and well-being vary within individuals as compared to across individuals? The inclusion of concurrent assessments of various potential correlates of mental health (e.g., physical activity, social support) will allow for dynamic models to better understand how mental health and its correlates are prospectively related to one another. Cross-lagged panel models, and newer modelling offshoots, can address whether one’s state-level or trait-level of mental health predicts subsequent levels of the correlate, or vice versa (Orth et al., 2021). The aim of such models is to provide insight about the prospective associations between mental health and its correlates, and ideally, to estimate causal associations between these variables.

### Next Steps

As the Canadian narrative around mental health has shifted, so has the impetus to invest in the future of mental health for older adults. The country has taken a more active role in ensuring that future generations of ageing Canadians receive the best possible care. This has been reflected in the ways in which funding has been allocated towards mental health research, the prioritisation of mental health in policy, and the public profile of mental health. For example, recently, the Public Health Agency of Canada (2022) announced a $12.2 million project directed specifically at mental health promotion. As more Canadians experience mental health challenges as they age, there will be an increasing need for innovative approaches to mental health research, intervention, and prevention. Working in concert with changes in law and policy the lived experience of mental ill-health in Canada is also shaped by ongoing research, albeit via different mechanisms.

## Conclusion

Ageing Canadians represent a greater proportion of the population than ever before. The mental health needs and challenges faced by this ever-evolving group is shaped by myriad factors unique to Canada, ranging from the political to geographic landscape. Over the course of Canadian history, the way the mental health of older adults has been addressed in the eyes of the law has shifted dramatically, having considerable impacts on the ways in which Canadians have lived with mental illness. Moving from paternalistic models to those in which equity and diversity are prioritised, the way the law has viewed the mental health of older adults has come a long way, but there is still much work to be done. To address the issues older adults face in Canada, longitudinal studies have been put in place to empirically investigate the ageing process. With world-leading ongoing longitudinal studies, such as the Canadian Longitudinal Study on Ageing, researchers in Canada are making long-term investments in the future of ageing Canadians. Although Canada is a relatively young country, it has a strong history of progression and innovation in working towards fostering the best mental health trajectories for ageing Canadians. Working to enact change in the laws and policies that govern mental health as well as developing better evidence as to the ways in which we can identify and treat mental illness it is hoped that positive change can be enacted in the lived experience of mental health in Canada for older adults.
